# A Critical Review of Methodologies for Evaluating Iron Fertilizers Based on Iron Reduction and Uptake by Strategy I Plants

**DOI:** 10.3390/plants13060819

**Published:** 2024-03-13

**Authors:** Alejandra Arcas, Sandra López-Rayo, Agustín Gárate, Juan J. Lucena

**Affiliations:** Department of Agricultural Chemistry and Food Science, Universidad Autónoma de Madrid, 28049 Madrid, Spain; alejandra.arcas@estudiante.uam.es (A.A.); sandra.lopez@uam.es (S.L.-R.); a.garate@uam.es (A.G.)

**Keywords:** iron deficiency, chlorosis, Strategy I, Fe chelate reductase, Fe uptake, ^57^Fe

## Abstract

Under iron (Fe)-limited conditions, plants have developed strategies for acquiring this essential micronutrient. Several Fe sources have been studied as potential fertilizers, with Fe synthetic chelates being the most used to prevent and correct Fe chlorosis in crops. The determination of the activity of the Fe chelate reductase (FCR) enzyme has long been described in the literature to understand the efficiency of Strategy I plants in acquiring Fe from fertilizers under deficient conditions. Other experiments have focused on the translocation of Fe to the plant to define the effectiveness of Fe fertilizers. Yet, both assays are relevant in knowing the capacity of a novel Fe source and other compounds alleviating Fe chlorosis in Strategy I plants. This work reviews the methodologies that are used in FCR assays to evaluate novel Fe fertilizers, including the factors modulating the results obtained for FCR assay activity, such as the Fe substrate, the Fe level during the growing period and during the FCR assay, the pH, the choice of an *in vivo* or *in vitro* method, and the plant species. A discussion of the benefits of the concurrence of FCR and Fe uptake assays is then presented alongside a proposed methodology for assessing the effectiveness of Fe fertilizers, emphasizing the importance of understanding chemical and physiological plant interactions. This methodology unifies key factors that modify FCR activity and combines these with the use of the ^57^Fe tracer to enhance our comprehension of the efficacy of Fe-based fertilizers’ effectiveness in alleviating Fe chlorosis. This comprehensive approach not only contributes to the fundamental understanding of Fe-deficient Strategy I plants but also establishes a robust method for determining the efficiency of novel sources for correcting Fe deficiency in plants.

## 1. Introduction

Iron (Fe) is one of the essential micronutrients for plants. Due to its chemical properties, Fe is involved in numerous redox reactions, playing a key role as a co-factor for enzymes in several processes such as respiration and being critical for photosynthesis and chlorophyll biosynthesis [1,2].

Although Fe is the fourth most abundant element in the Earth’s crust, it is scarcely available for plant nutrition in areas of calcareous and/or alkaline soils [3], being mainly precipitated in the form of Fe hydroxides. Iron deficiency in plants is characterized by the yellowing of interveinal young leaves, known as Fe chlorosis [4], which is responsible for a nutritional disorder causing a metabolic imbalance and ultimately leading to a reduction in crop yields [2].

Under Fe-limited growth conditions, higher plants have developed different responses for adaptation, grouped into two main strategies: Strategy I and Strategy II [5]. Dicotyledonous and no-grass monocotyledonous, such as cucumber and *Arabidopsis*, use Strategy I (a reduction-based strategy), while grasses use Strategy II (a chelation-based strategy). Strategy I holds special importance in agriculture, with most horticultural and fruit crops employing it. Strategy I responses consist of a complex set of reactions that solubilize unavailable Fe, improve its uptake, and favor its translocation into the plant (for recent reviews, see [2,6,7,8,9]). Strategy I entails a reduction-based process including several steps. First, there is an excretion of protons from the roots to the rhizosphere to increase the solubility of Fe compounds. The acidification of the rhizosphere is carried out by a plasma-membrane-bound H^+^-ATPase [10,11]. In the case of *Arabidopsis*, the *AHA1* gene encodes it [11]. This step also includes the excretion of different compounds, including phenolics, such as carboxylics, coumarins, or flavins, to increase Fe solubilization [12]. Secondly, the reduction of Fe(III) compounds to Fe^2+^ occurs in the root surface, carried out by the Ferric chelate reductase (FCR) enzyme [13]. Finally, the uptake of Fe^2+^ takes place through a plasma membrane transport system [14]. Ferrous ion is transported across the root epidermal membrane cells by a high-affinity transporter [6,15]. The dominant genes encoding for ferric reductase and ferrous transporters were first identified in *Arabidopsis* in the 1990s; the FCR enzyme is encoded by the ferric chelate reductase oxidase gene *FRO2*, and the ferrous iron transporter is encoded by the iron-regulated transporter gene *IRT1* [10,11].

Several approaches have been used to prevent and/or correct Fe deficiency. Currently, the application of Fe fertilizers is the most common solution for established crops [16,17] that are grown on calcareous soils. Fe chelates are the most effective due to their high stability in soils, but Fe complexes (mainly from natural sources), inorganic soluble salts (such as sulfates), slow-release Fe synthetic organic or mineral products, or formulations that promote Fe solubilization in soils, such as micro-organism-based formulations [18] are also used.

The study of the efficiency of novel Fe fertilizer formulations has included the development of different methodologies based on analytical chemistry and plant physiology. One of the most widely studied is the FCR (or FeCR) activity [17,19,20,21,22,23,24,25,26,27,28,29]. This enzyme reduces any Fe(III) bond to a ligand in the rhizosphere (as Fe chelate, or Fe complex, synthetic or natural) to Fe^2+^. The effectiveness of reduction depends on several factors such as the plant species and the Fe status of the plant, as well as the Fe chemistry (mostly chelates) in the soil or growing medium [30]. Some studies have shown that FCR reduces more Fe than that uptake by the roots [19,26], and consequently, the FCR activities that were determined in some Fe chelates did not correlate well with their efficacy to alleviate chlorosis as determined through leaf chlorophyll index or plant biomass [25,31]. Furthermore, most of the FCR assays described in the literature use excised apical roots, the enzyme activity is induced *in vitro*, and only a few studies are conducted by submerging roots of intact plants. In a previous review, Abadía et al. [30] pointed out some of these aspects and questioned the FCR test to assess the effectiveness of Fe fertilizer, suggesting the development of alternative tests.

Current studies on Fe acquisition focus on the molecular regulation and biochemical mechanisms related to plant responses to Fe deficiency [2,6,7,8,9,32,33,34,35] and do not pay much attention to the importance of the Fe speciation to modulate FCR activity or the use of roots of intact plants.

In light of this, the present review examines the latest analytical methodologies by using FCR assays to evaluate the efficacy of novel Fe chelates with an emphasis on factors that can modulate the FCR activity, and in particular, on the chemical nature of Fe chelates. Finally, a detailed methodology is proposed to evaluate Fe chelates and other Fe fertilizers based on the literature findings on Fe reduction versus Fe uptake.

## 2. FCR as a Tool to Study Fe Source Effectiveness in Correcting Fe Chlorosis

The main studies that use FCR assays as a tool to evaluate the effectiveness of new Fe sources in recent years are summarized in Table 1 (2016–2023). They include the FCR methodology applied to the study of the plant response to new Fe sources, different crop varieties, and the Fe status in plants subjected to different stresses. The list of abbreviations used in the works cited in Table 1 is presented in Table 2.

### 2.1. Fe^2+^ Trapping Agent and Fe-Chelate Substrate for the FCR

The analytical methodology used to determine this enzyme capacity is based on the addition of a specific Fe^2+^ trapping agent to the rhizosphere. Bathophenanthroline disulfonic acid (BPDS) or ferrozine are the most used ligands due to their high affinity (formation logK^0^ = 20.2, for Fe(II)(BPDS)_3_ chelate and logK^0^ = 15.7 for Fe(II)(Ferrozine)_3_ [36]) and the strong red color formed (ε = 22,140 M^−1^ cm^−1^ at 535 nm for Fe(II)(BPDS)_3_ and 27,900 M^−1^ cm^−1^ at 562 nm for Fe(II)(Ferrozine)_3_ [37]). Once the Fe(III) is reduced to Fe^2+^ by the FCR, it is immediately chelated by these ligands, and its concentration is determined through colorimetric methods. In fact, this method was initially used to demonstrate the existence of a reduction-based iron uptake in plants [30,38].

**Table 1:** Summary of main published works in recent years (2016–2023) where FCR assay is used as an indicator of the plant response to treatments to correct Fe chlorosis. The table indicates the Fe source used as a substrate for the FCR enzyme, pH conditions and used buffer, plant species, treatment target of the study, type of root sample (*in vivo* with the whole plant or *in vitro* with excised root segments), sampling times and Fe^2+^ trapping agent used.

FCR Substrate	pH	Buffer	Plant Species	Treatments Studied to Correct Fe Chlorosis	Type of Root Sample	Measured Time	Trapping Agent	Reference
EDTA/Fe^3+^	5.5	MES	Soybean (*Glycine max* cv. Williams 82)	EDDHA/Fe^3+^; Fe(mpp)_3_.	Whole plant	45 min	BPDS	[17]
EDTA/Fe^3+^	6.5	Tris	Pear (*Pyrus communis* cv. Deveci on OHF-333 (Old Home x Farmingdale) and BA-29 (*Cydonia oblonga* Mill.) rootstocks	PGPR: *Alcaligenes* 637Ca, *Agrobacterium* A18, *Staphylococcus* MFDCa1, MFDCa2, *Bacillus* M3 and *Pantoea* FF1; FeSO_4,_ EDDHA/Fe^3+^	Excised roots	180 min	BPDS	[39]
EDTA/Fe^3+^	5.0	MES	Tomato (*Lycopersicon esculentum* Mill.Seny F1)	*o,o*EDDHA/Fe^3+^ formulations: meso and d,l-racemic *o,o*EDDHA/Fe^3+^	Whole plant	30 min	Ferrozine	[40]
*o,o*EDDHA/Fe^3+^, EDTA/Fe^3+^, IDHA/Fe^3+^, EDDS/Fe^3+^, Spruce and Eucalyptus LS/Fe^3+^, LN/Fe^3+^ and GA/Fe^3+^	6.0	MES	Soybean (*Glycine max* cv. Klaxon)	*o,o*EDDHA/Fe^3+^, EDTA/Fe^3+^, IDHA/Fe^3+^, EDDS/Fe^3+^, Spruce and Eucalyptus LS/Fe^3+^, LN/Fe^3+^ and GA/Fe^3+^	Whole plant	0,10, 20 and, 60 min	BPDS	[25]
EDTA/Fe^3+^	6.5	Tris	Peach (*Prunus persica* L cv. Elegant Lady on GF 677 and Nemaguard rootstocks)	PGPR: *Alcaligenes* 637Ca, *Agrobacterium* A18, *Staphylococcus* MFDCa1, MFDCa2, Bacillus M3 and *Pantoea* FF1	Excised roots	180 min	BPDS	[41]
EDTA/Fe^3+^	-	Tris	Apple (*Malus domestica* cv. Braeburn on M9 and MM106 rootstocks)	PGPR: *Alcaligenes* 637Ca, *Agrobacterium* A18, *Staphylococcus* MFDCa1, MFDCa2, *Bacillus* M3 and *Pantoea* FF1; EDDHA/Fe^3+^	Excised roots	180 min	BPDS	[42]
EDTA/Fe^3+^	5.5	MES	Cucumber (*Cucumis sativus* L. cv. Ashley)	FeSO_4_, EDTA/Fe^3+^, HG/Fe^3+^, EDDHA/Fe^3+^	Excised roots	30 min	BPDS	[43]
*o,o*EDDHA/Fe^3+^, EDTA/Fe^3+^, azotochelin/Fe^3+^, DPH/Fe^3+^	7.5	HEPES	Cucumber (*Cucumis sativus* L., cv. Ashley)	*o,o*EDDHA/Fe ^3+^, EDTA/Fe^3+^, azotochelin/Fe^3+^, DPH/Fe^3+^	Whole plant	0, 10, 20, 60, and 120 min	BPDS	[26]
EDTA/Fe^3+^ and LN	6.0	MES	Cucumber (*Cucumis sativus* L. cv. Ashley)	EDTA/Fe^3+^, LN/Fe^3+^	Whole plant	0, 10, 20, 60, and 120 min	BPDS	[44]
EDTA/Fe^3+^	5.5	-	Purple-fleshed sweet potato (*Ipomoea batatas* (L.) Lam.) varieties xuzi8 and xuzi6	FeSO_4_, Fe_2_(SO_4_)_3_, EDTA/Fe^3+^	Excised roots	30 min	Ferrozine	[45]
EDTA/Fe^3+^	5.5	MES	Quince (*Cydonia oblonga* Mill cv. Isfahan)	PGPR: *Pseudomonas fluorescens* and *Microccucuce yunnanensis*; EDDHA/Fe^3+^	Excised roots	60 min	Ferrozine	[46]
EDTA/Fe^3+^	5.5	MES	Cucumber (*Cucumis sativus* L. cv. Jinyan No.4)	GABA, EDTA/Fe^3+^	Excised roots	60 min	Ferrozine	[47]
EDTA/Fe^3+^	5.5	MES	Alfalfa (*Medicago sativa* L. cv. Vernal)	AMF: *Glomus intraradices*, *Glomus mosseae*, *Glomus aggregatum, Glomus etunicatum*; EDTA/Fe^3+^	Excised roots	20 min	Ferrozine	[48]
EDTA/Fe^3+^	5.5	MES	Tomato (*Solanum lycopersicum* L. cv. AKRAI F1) and cucumber (*Cucumis sativus* L. cv. EKRONF1)	Legume-derived protein hydrolysate, EDTA/Fe^3+^	Whole plant	20 min	BPDS	[49]
*o,o*EDDHA/Fe^3+^, EDTA/Fe^3+^ and Fe-heme	6.0 and 7.5	MES; HEPES	Cucumber (*Cucumis sativus* L. cv. Ashley)	o,oEDDHA/Fe^3+^, EDTA/Fe^3+^, powder formulation derived from bovine-blood (Fe-heme)	Whole plant	0, 10, 20, 60, and 120 min	BPDS	[27]
EDTA/Fe^3+^	6.0	MES	Strawberry (*Fragaria x ananassa* Duch. cv. “Diamond”)	Gramineous plant extract, Fe-EDDHA/Fe^3+^	Excised roots	60 min	BPDS	[50]
EDTA/Fe^3+^	5.5	MES	Soybean (*Glycine max* cv. “Williams 82”)	3,4-HPO/Fe^3+^: Fe(mpp)_3_, Fe(dmpp)_3_, Fe(etpp)_3_	Whole plant	45 min	BPDS	[24]
PDMA/Fe^3+^, EDTA/Fe^3+^ and Cit/Fe^3+^	7.0, 8.0 and 9.0	PIPES, EPPS and CHES	Cucumber (*Cucumis sativus* L. cv. ‘Hokushin’)	PDMA/Fe^3+^, Cit/Fe^3+^, EDTA/Fe^3+^	Whole plant	60 min	BPDS	[28]
EDTA/Fe^3+^	6.0	MES	Strawberry (*Fragaria × ananassa* Duch. cv. ‘Portola’)	Microorganism-based formulations (MBF): an inoculum (In) composed of: organic matter, *Glomus* spp., rhizosphere bacteria, *Trichoderma*, *Streptomyces* spp. and *Trichoderma* spores; *o,o*EDDHA/Fe^3+^, EDDHA/Fe^3+^	Excised roots	60 min	BPDS	[18]
EDTA/Fe^3+^	5.5	MES	Quince (*Cydonia oblonga* Mill cv. Isfahan)	Arbuscular mycorrhizal (AM) fungi: *Funneliformis mosseae* and *Rhizophagus intraradices;* EDDHA/Fe^3+^	Excised roots	60 min	Ferrozine	[51]
EDTA/Fe^3+^	5.5	MES	Soybean (*Glycine max* cv. Williams 82)	PGPR: inoculation with S. *fuliginis* ZR 1–6, inoculation with P. *jessenii* ZR 3–8. No Fe source added	Whole plant	45 min	BPDS	[52]
EDTA/Fe^3+^	5.3	-	Japanese rowan (*Sorbus commixta*)	FeSO_4_, EDTA/Fe^3+^, DTPA/Fe^3+^	Whole plant	20 min	2,2‘-bipyridyl	[53]
EDTA/Fe^3+^	5.5	MES	Chinese crab apple (*Malus hupehensi*)	Brassinolide (BL), EDTA/Fe^3+^	Excised roots	120 min	Ferrozine	[54]
EDTA/Fe^3+^	6.0	MES	Strawberry (*Fragaria x ananassa* Duch. cv. “Diamond”)	Organic acids: citric acid (CA), malic acid (MA), and succinic acid (SA); EDDHA/Fe^3+^	Excised roots	60 min	BPDS	[55]
EDTA/Fe^3+^	6.0	MES	Cucumber (*Cucumis sativus* L. and Viridis F1 hybrid)	H_2_bpcd/Fe^3+^, EDTA/Fe^3+^	Excised roots	20 min	Ferrozine	[29]
EDTA/Fe^3+^	5.5	MES	Cucumber (*Cucumis sativus* L. cv Chinese long)	Fe-biochelate containing vegetal-derived peptides, EDDHA/Fe^3+^	Excised roots	30 min	BPDS	[16]
EDTA/Fe^3+^	6.00	MES	Cucumber (*Cucumis sativus* L. cv. Joker)	NH, NFH, Cit/Fe^3+^, EDDHA/Fe^3+^	Excised roots	15 min	BPDS	[56]
EDTA/Fe^3+^	6.00	MES	Cucumber (*Cucumis sativus* L. cv. Joker F1)	NH, NFH	Excised roots	15 min	BPDS	[57]

The common thread across all studies is that FCR is activated in the roots by the presence of an Fe source in the rhizosphere as a mechanism for its subsequent uptake while the methodology applied may differ. Some studies were conducted *in vivo* by removing plants from the growing media and submerging roots in an aerated solution with the corresponding sequestering ligand [17,24,25,26,27,28,40,44,49,52,53]. Other studies use excised root segments from the plants, called *in vitro*, and similarly these root samples are immersed in solutions [16,18,29,39,41,42,43,45,46,47,48,50,51,54,55,56,57]. The Fe substrate for the FCR can also differ. For most of the assays described in the literature over the past few years (listed in Table 1), the FCR assay was conducted after the application of an Fe pretreatment, or after a modification to the rhizosphere. This test serves as an indicator of the nutritional status of the Fe plant or the response to induced stress. In both cases, the study of FCR activity is carried out using EDTA/Fe^3+^ as an FCR substrate (Table 1). However, other studies have assessed how plants reduce and uptake Fe from new Fe sources and use these Fe sources as FCR substrates (see Section 3).

**Table 2:** List of abbreviations used for the FCR substrates, treatments and buffers used in Table 1.

Abbreviation	Full Name
[S,S]-EDDS	S,S-isomer of the ethylenediaminedisuccinate
3,4-HPO	3-hydroxy-4-pyridinone
AM	Arbuscular mycorrhizal
Azotochelin	(N, N′)-2,6–Bis(2,3-dihydroxybenzoyl)-L-lysine]
BPDS	Bathophenanthroline disulfonic acid
CHES	N-cyclohexyl-2-aminoethanesulfonic acid
Cit	Citrate
DCHA	2-(2-((2-hydroxy- benzyl) amino)ethylamino)-2-(2-hydroxyphenyl)acetic acid
DPH	N-Dihydroxy-N,N′-diisopropylhexanediamide
DTPA	Diethylenetriaminepentaacetic acid
EDDHSA	N,N′-Ethylenediamine-di-(2-hydroxy-5-sulfophenylacetic acid)
EDDS	Ethylenediaminedisuccinic acid
EDTA	Ethylenediaminetetraacetic acid
EPPS	3-[4-(2-hydroxyethyl)- 1-piperazinyl]propanesulfonic acid
Fe-heme	Powder formulation derived from bovine-blood
FeSO_4_	Fe sulfate
GA	Gluconate
GABA	Gamma-aminobutyric acid
H_2_bpcd	N,N′-bis(2-pyridylmethyl)-*trans*-1,2- diaminocyclohexane N,N′-diacetate
HEPES	4-(2-hydroxyethyl)-1-piperazine ethanesulfonic acid)
HBED	N,N′- bis(2-hydroxybenzyl)ethylenediamine-N,N′-diacetic acid;
Hdmpp	3-hydroxy-1,2-dimethylpyridin-4(1H)-one
Hetpp	2-ethyl-3-hydroxypyridin-4(1H)-one
HJB	N,N′-Bis(2-hydroxy-5-methylbenzyl) ethylenediamine-N,N′-diacetic acid
HG	Heptagluconate
Hmpp	3-hydroxy-2-methylpyridin-4(1H)-one
IDHA	N-(1,2-dicarboxyethyl)-D,L-aspartic acid
LN	Leonardite
LS	Lignosulfonate
MES	2-(N-morpholino)ethanesulfonic acid
MBF	Microorganism-based formulations
mpp	2-methyl-3-hydroxy-4-pyridinonate
NFH	Nanoferrihydrite
NH	Nanohematite
*o,o*EDDHA	ethylenediamine-N-N′bis(o-hydroxyphenylacetic) acid
*o,p*EDDHA	Ethylenediamine-N(o-hydroxyphenylacetic)-N′(p-hydroxyphenylacetic) acid
PIPES	Piperazine-1,4-bis(2-ethanesulfonic acid)
PDMA	Proline-2′-deoxymugineic acid
PGPR	Plant growth-promoting rhizobacteria

### 2.2. pH Influence on FCR Activity

In papers where EDTA/Fe^3+^ is used as the FCR substrate, pH values were generally set between 5 and 6 (Table 1). These conditions were already known to be optimal to achieve the maximum FCR activity while FCR rates decrease at higher pH [58]. This is why many studies on Fe nutrition are carried out at this pH [59], where the best nutrient absorption and, therefore, plants’ responses are found in field crops [17,24]. However, Fe chlorosis in crops occurs commonly in calcareous soils where pH is in the range of 7.4–8.5 [60]. Moreover, the presence of high bicarbonate content and high pH decreases Fe uptake by the plant. Waters et al. [59] observed that an increase in bicarbonate concentration in the nutrient solution caused significant differences in FCR activity between the plants tolerant to Fe chlorosis at two Fe levels (low level and sufficiency). The activity was higher at low Fe levels, although this increase in FCR rates did not seem enough to maintain Fe uptake. Additionally, they observed that the stress caused by the alkalinity of the medium and the limitation of Fe supply led to an overexpression of classical iron uptake genes such as *FRO2*, *IRT1*, or *AHA2* [59]. Di Foggia et al. [27] evaluated FCR activity in cucumber plants at two different pH values: 6 and 7.5. The authors pointed out that at pH 7.5, all Fe sources exhibited a lower amount of reduced Fe compared to those observed at pH 6. However, even at a buffered pH of 7.5, a pH value close to the typical pH of calcareous soil, FCR activity can be determined [19,26]. Ueno et al. [28] also evaluated the reduction of Fe(III) under neutral to alkaline pH conditions (7.0, 8.0, and 9.0) obtaining a similar result: FCR activity declines as the pH increases, but it remains active.

### 2.3. Fe Status, Fe Substrate, and Other Environmental Factors in the Growing Media

An efficient fertilizer for the remediation of Fe chlorosis should act in response to at least one of the factors responsible for the plant Fe deficiency: the low solubility of Fe^3+^; the low presence of Fe species in solution, e.g., Fe(OH)_x_^3−x^, Fe-mineral and Fe-organic complexes; the slow solubilization rate of Fe mineral phases; the impaired reduction and/or uptake mechanisms; and the limited Fe translocation into the plant. Most of the fertilizers evaluated through the FCR activity test were Fe chelates and complexes [16,17,24,25,29], but Fe siderophores [26,28], and plant-growth-promoting rhizobacteria (PGPR) [39,42,52] have also been studied using this methodology.

In some assays, FCR activity increased when Fe-deficient plants were resupplied with Fe and the enzyme activity decreased over time as the plant reached Fe-sufficiency [30]. The FCR rates depend on several factors as described above, such as nutritional status, pre-treatment of plants with Fe sources, growth in hydroponic or soil conditions, pH, etc. Some authors have pointed out that a decrease in the rate of FCR activity is an indicator of plant recovery from Fe chlorosis [18]. Stressed strawberry plants showed an increase in FCR activity after plants were grown without Fe application compared to when treatments were applied. The reduced FCR rates were consistent with a reduction in chlorosis symptoms [18]. Fuentes et al. [43] reported a reduction in FCR root activity levels in chlorotic cucumber plants treated by foliar Fe application. Similarly, Xiao et al. [53] obtained higher FCR rates in Fe-deficient plants than in plants growing in a medium with Fe treatments. On the other hand, there are other experiments where FCR activity in chlorotic plants without Fe supplementation showed lower FCR activity ratios than in plants with treatments to alleviate chlorosis. Santos et al. [24] showed that soybean plants with no Fe added exhibited lower FCR activity than plants with Fe treatments in hydroponic culture. They propose that these results may be a consequence of the enzyme needing Fe for its activation. Similarly, chlorotic strawberry plants with no Fe exhibited lower FCR rates compared to those with sufficient Fe. This difference may be attributed to the rapid growth characteristic of this cultivar [55,61].

In summary, FCR is linked to the nutritional status of the plant, so this assay is a useful tool for evaluating the overall Fe requirements of the plant. Thus, Fe status must be carefully established when using the FCR assay to compare treatments. To avoid the contradictory effect when plants were pretreated without Fe, Lucena and Chaney [19] found that pretreatment doses as low as 5µM Fe were the most adequate concentrations to obtain stressed cucumber plants and produce satisfactory FCR rates. Plants in a normal agronomic soil culture have a certain amount of Fe available, even if it is not sufficient. Therefore, the addition of a low dose of Fe in the growing media for the evaluation of treatments to correct Fe deficiency seems a more realistic condition to evaluate Fe products.

Micro-organisms and other biostimulant formulations themselves do not provide Fe to plants but they have been shown to produce an increase in FCR activity which can contribute to alleviating Fe chlorosis [41,42,47,51,52]. For instance, İpek et al. [39] reported that root inoculation with rhizobacteria increased FCR rates and Fe availability through the excretion of organic acids to the rhizosphere and decreased soil pH.

### 2.4. Diversity of Cultivar Responses to FCR Activity

FCR is strongly dependent on the crop used [31]. Most of the works used cucumber (*Cucumis sativus* L.) as a model horticultural plant in the evaluation of treatments to alleviate Fe chlorosis. It is a species that is well known for its efficiency in activating Fe acquisition mechanisms under limited conditions of this micronutrient. To carry this out, the FCR enzyme activity increases especially at pH 6 which are the conditions applied to most of the studies described in the literature. In addition, several authors have used the determination of the FCR to test the efficiency of the cultivars against Fe chlorosis. Jolley et al. [62] concluded that the FCR assay was potentially reliable for evaluating soybean genotypes for resistance to Fe chlorosis and could distinguish highly resistant genotypes better than in-field assays. Similar results were obtained for grapevine rootstocks [63] and quince, pear, and olive [64]. However, Nadal [65], found a better relationship between the efficiency of soybean cultivars with a complete labeled Fe uptake assay instead of the FCR assay using the same experimental and growth conditions which that be discussed later (see Section 4).

## 3. Iron Chelates as FCR Substrates

A common practice in agriculture is the use of synthetic chelates to prevent and correct Fe chlorosis. An efficient Fe chelate should be stable enough to maintain Fe in solution (or soil solution) but must also be able to release that Fe to plants [24]. The reduction rate of chelate-bound Fe(III) to release Fe^2+^ in a solution depends on the chemical properties of the chelating agent and the environmental conditions. The most commonly used chelate for FCR assays (Table 1) is EDTA/Fe^3+^ because of its known use in agriculture and the high rates of FCR activity found for this chelate. In stressed cucumber plants at equivalent pH conditions (pH 6 [27] and pH 7.5 [26,27]), the reduction rate obtained by FCR using EDTA/Fe^3+^ is higher than when using the highly stable and efficient *o,o*EDDHA/Fe^3+^.

Therefore, the question arises as to whether it is feasible to establish a correlation between the reduction obtained in the FCR test and the effectiveness of the Fe source in plants. As discussed in this review, numerous factors impact the rate of Fe reduction by FCR, and these factors may not necessarily be aligned with an efficient acquisition of Fe by the plant.

### 3.1. Relationship between FCR and the Chemical Fe-L Stability

**Figure 1:**
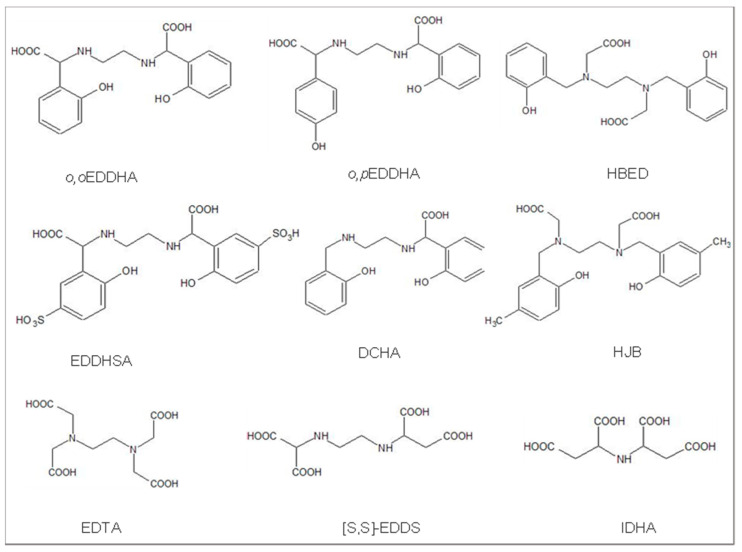
Structures of the main synthetic chelating agents studied as FCR substrates and described in Table 2.

In 2006, Lucena and Chaney [19] compared different Fe chelates and concluded that the best substrates for FCR activity in slightly chlorotic cucumber plants were Fe chelates with lower stability, despite being less efficient in alleviating Fe chlorosis. Table 3 presents a summary of the thermodynamic stability constants (logK^0^_ML_) for selected synthetic Fe chelates used as substrates for FCR assays in the literature, along with the corresponding references for the publications investigating reduction assay methodologies. The chemical structures of the ligands mentioned in Table 3 are shown in Figure 1. The most used chelate, *o,o*EDDHA/Fe^3+^, exhibited a lower FCR rate than other chelates despite being one of the most widely used chelates to alleviate chlorosis in plants. This may be related to its high stability constant.

Nadal et al. [21] compared hexacoordinated phenolic chelates such as HBED/Fe^3+^, *o,o*EDDHA/Fe^3+,^ and HJB/Fe^3+^ as Fe substrates for FCR in Fe-deficient cucumber plants. Although HBED/Fe^3+^ has a higher stability constant (Table 3) than *o,o*EDDHA/Fe^3+^, similar Fe reduction rates were found for FCR activity. Furthermore, Lucena and Chaney [19] did not observe differences between the FCR rate with *o,o*EDDHA/Fe^3+^ and HBED/Fe^3+^ in Fe-deficient cucumber plants. However, when hexacoordinated non-phenolic chelates such as the structural isomers EDTA/Fe^3+^ and [S,S′]EDDS/Fe^3+^ were used as FCR substrates in chlorotic soybean plants, significant differences were obtained [25]. In this case, the differences were related to their chemical stability, and the [S,S´]EDDS/Fe^3+^ has a lower stability constant (Table 3) which correlated to its greater Fe reduction rates by FCR in comparison to EDTA/Fe^3+^.

When *o,o*EDDHA/Fe^3+^ stereoisomers have been compared as FCR substrates, the mesoEDDHA/Fe^3+^ exhibited greater rates than racEDDHA/Fe^3+^ [19,68], with the racemic mixture being the most stable of both isomers (Table 3). Lucena and Chaney [19] suggested that when the EDDHA/Fe^3+^ (mixture of both isomers) was used, the mesoEDDHA/Fe^3+^ isomer was firstly reduced by the FCR. In hydroponics assays, formulations containing a mixture of isomers of EDDHA/Fe^3+^ have been shown to result in preferential depletion of the meso-isomer from nutrient solutions [40], and the Fe provided by the meso isomer is preferentially absorbed [68].

In the case of EDTA/Fe^3+^, greater reduction rates by FCR have been reported compared to *o,o*EDDHA/Fe^3+^, but EDTA/Fe^3+^ has been demonstrated to be less efficient than *o,o*EDDHA in alleviating Fe chlorosis [19,27]. The different FCR rates observed between the two chelates could be due to the complexation of the reduced Fe species by EDTA. The EDTA/Fe^2+^ (logK^0^ 14.94 [71]) chelate is much more stable than the EDDHA/Fe^2+^ chelate (logK^0^ 5.3 [72]) [19]. Thus, it is reasonable to think that the formation of EDTA/Fe^2+^ is thermodynamically favorable, and then, the Fe^2+^ stabilized by EDTA is less available for the subsequent root uptake. EDTA acts as an Fe^2+^ trapping agent when Fe-EDTA is used to provide Fe to the plants. In fact, the BPDS ligand is used in FCR assays as the trapping reagent because of its high Fe^2+^ chelate formation constant (logK^0^ = 20.28 [73]).

Weger et al. [74] concluded that the stronger the Fe chelates, the lower the rates of FCR activity, suggesting that a strong chelating agent could chelate also the Fe^2+^, avoiding the Fe^2+^ uptake by *Chlorella kessleri* algae. The fact is that the strong Fe^3+^ chelators are not relevant strong Fe^2+^ chelators, so the explanation should be better related to the forced reoxidation of the Fe^3+^ by the stronger chelators to form Fe^3+^ chelates [23].

Thus, the chemical stability of the Fe(III) chelates does not completely explain the differences found in the Fe reduction by roots.

### 3.2. Influence of the Geometry of Fe Chelates on FCR Rate

The geometry of the Fe chelate and the number of electron-donating groups of the ligand also seem to be determinants in the access of the FCR enzyme to the chelated Fe^3+^, which is not necessarily correlated to the stability constant. It has been reported that an octahedral closed geometry such as that of *o,o*EDDHA/Fe^3+^ hinders the accessibility of the enzyme to Fe^3+^. More open structures, as in the case of the *o,p*EDDHA/Fe^3+^, with the p-hydroxy phenolate not binding the Fe allows a fast interaction of the chelate with the FCR active center, resulting in a greater FCR rate [66]. When comparing the FCR rate with *o,p*EDDHA/Fe^3+^ as substrate with other open structure Fe chelate DCHA/Fe^3+^, and *o,o*EDDHA/Fe^3+^ [22], the Fe reduction rate was higher with *o,p*EDDHA/Fe^3+^, followed by DCHA/Fe^3+^, and the lowest reduction was found for *o,o*EDDHA/Fe^3+^. Thus, the low FCR rate obtained in the experiments for the *o,o*EDDHA/Fe^3+^ chelate would be explained not only by its high stability (Table 3) but also by its closed structure with six donor groups that bind to Fe. The *o,p*EDDHA/Fe^3+^ and DCHA/Fe^3+^ have similar stability constants (Table 3) and only five donor groups bind Fe in a pentacoordinated structure, making these better FCR substrates than *o,o*EDDHA/Fe^3+^ [22,66]. Escudero et al. [23] pointed out the relationship between the chemical properties of the Fe chelate and the way they interact with FCR.

### 3.3. Other Chemical Properties of the Fe Chelates That Influence the FCR

Other chemical characteristics of Fe chelates must be considered, such as the polarity or the electrochemical properties [23] or the charge of the chelate [75]. Chaney [75] pointed out that the charge of the chelate is important, in addition to its stability. Although EDDHSA/Fe^3+^ has a stability constant similar to meso *o,o*EDDHA/Fe^3+^ (Table 3), the reduction obtained by the FCR was much lower [19]. The authors suggested that the three negative charges of the EDDHSA/Fe^3+^ chelate might be related to the low rate of enzyme activity. Moreover, the EDDHSA/Fe^3+^ used in that work was a mixture of isomers containing polycondensated compounds [76]. According to several authors, polymers such as Fe humates [44], a powder formulation derived from bovine blood (Fe-heme) [27] and Fe lignosulfonates [67], yield a lower FCR than synthetic chelates. In these compounds, the large structures, with the multiple negative charges of their binding groups, may impair the accessibility to the reduction sites by the FCR. This may also apply to the EDDHSA/Fe^3+^ polycondensates.

Upon comprehensive review of all the results, it is evident that the differences in the FCR assay can be attributed to numerous factors, including methodological aspects but also the different chemical and structural properties of Fe sources, and the efficiency of the FCR appears not to be necessarily correlated with the effective Fe uptake by plants.

## 4. Iron Acquisition by Roots: Insights from FCR Assay

### 4.1. Iron Reduced vs. Iron Uptake

All steps in plant Fe acquisition—not just the FCR—must be considered when assessing Fe sources to address Fe deficiency [30].

Additional processes have been described affecting the acquisition of exogenous Fe by plants in nutrient and soil solutions. Lindsay and Schwab [60] proposed for the first time that once the plant reduces Fe from Fe chelates, the chelating agent can solubilize other Fe naturally present in the soil. This mechanism, later called the ‘shuttle effect’, has not been fully demonstrated yet [77].

Once the reduction of Fe^3+^ to Fe^2+^ takes place in Strategy I plants, the Fe^2+^ needs to be transported from the rhizosphere to the root by IRT1 (Fe root transporter) [6]. However, some studies have shown that the amount of reduced Fe is much higher than the amount of Fe found in plants [65]. In fact, only 1.1% of the reduced Fe at pH 6 reached the xylem sap, and even a smaller fraction was determined at pH 7.5 (0.6%) in stressed cucumber plants [19,20]. Similarly, Orera et al. [68] compared the reduction rates and Fe uptake from meso and racemic *o,o*EDDHA/Fe^3+^ in longer experiments with sugar beet with Fe tracers at pH 6, concluding that only around 13% of the Fe reduced for the meso isomer and 50% for the racemic isomer were found in the whole plant. As discussed above, both the structure and the chemical properties of the Fe chelate have been demonstrated to be important factors affecting the reduction of Fe by the FCR, but they seem to also impact Fe assimilation by modulating the efficiency of the transporter (IRT) across the root plasma membrane [22].

Chaney and Bell [78] reported that almost all the Fe reduced from DTPA/Fe^3+^ reached the shoot, reaching even 100% in stressed peanut plants. However, this experiment was conducted without pH buffering, resulting in pH values around 4.0. Considering that the optimal pH for the FCR is 6.0, the actual Fe reduction was much lower than the optimal capacity of the enzyme and closer to the Fe uptake.

As previously discussed, the strong complexation of Fe^2+^ and the possibility of Fe^2+^ reoxidation suggest that FCR assays alone may not be adequate for determining the efficacy of Fe treatments to correct Fe chlorosis.

### 4.2. Stable Iron Isotopes as Tracers for FCR and Fe Uptake Studies

Since it has been shown that the plants absorb only a fraction of the reduced Fe, other assays that consider Fe uptake, translocation, and accumulation in the plant might be preferable. For instance, determining the Fe concentration in the xylem sap [19,65] and estimating the amount of Fe distributed in the different organs of a plant seem to be more appropriate tools. In line with this understanding, Rodríguez-Castrillón et al. [79] developed a method to track the Fe application through different plant organs using stable isotope ^57^Fe labeled fertilizers. The methodology includes the mathematical deconvolution process to discern between the Fe in plants coming from the labeled fertilizer and the one coming from natural sources (i.e., seed, soil, growing media). As an example, and thanks to the use of this methodology, a faster ability of *o,o*EDDHA/Fe^3+^ to provide Fe to soybean plants grown in calcareous soils, in comparison to the longer-lasting effects of the chelate HBED/Fe^3+^ has been demonstrated, predicting also the best doses and timing for application [80]. Additionally, with this technique, López-Rayo et al. [81] demonstrated that [*S,S*]-EDDS/Fe improved the Fe translocation from soil to leaves compared to other Fe chelates and pointed out the formation of Fe complexes to degradation products. Orera et al. [68] applied an isotopic methodology using a dual-stable Fe isotope tracer (^54^Fe and ^57^Fe) supplied to Fe-deficient sugar beet plants grown hydroponically. They determined the Fe distribution in plants 3 and 6 hours after the application of Fe-labeled treatments, showing differences between Fe treatments and concluding that plants did not discriminate between the two Fe isotopes.

Thus, using stable Fe isotopes as tracers is a useful methodology for evaluating the effectiveness of Fe fertilizers, especially in soils where the Fe natural sources cannot be discriminated by the analysis of the total Fe in plants.

## 5. Proposed Methodology to Evaluate the Efficiency of Novel Treatments to Provide Fe to Strategy I Plants

When an FCR assay is used to evaluate new Fe fertilizers or formulations designed to alleviate Fe chlorosis, it must be considered that FCR assays are dependent on the Fe substrate used, the Fe level during the growing period and during the FCR, the pH of the growing media, the choice of the *in vivo* or *in vitro* method, and the plant species are relevant. Furthermore, it has been proved that there is no consistent correlation between the FCR rate and the Fe uptake and Fe translocation to the plant. Based on this knowledge and the reviewed experiments, a concrete and well-described methodology is proposed to be applied in the study of the efficiency of novel sources to supply Fe to plants and to alleviate Fe chlorosis under controlled conditions.

**Figure 2:**
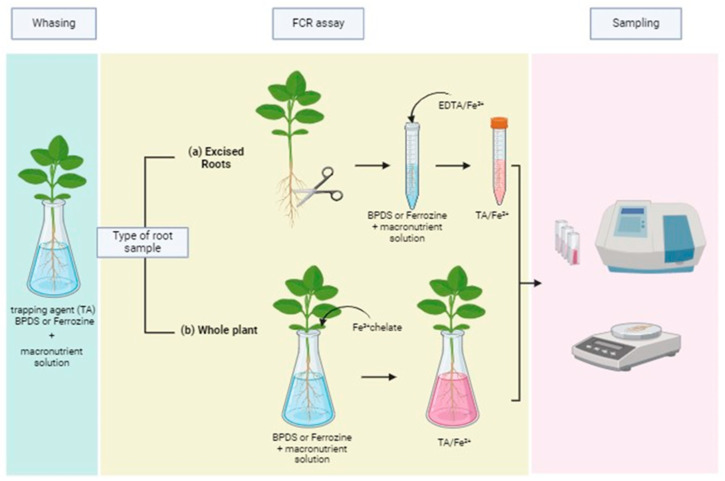
Schematic design of the methodological steps employed in FCR assays using excised roots (*in vitro*) or intact roots with the whole plant (*in vivo*). Created with BioRender.com.

Firstly, Fe status during the growth period is important. Plants should be developed in media with limited Fe but not in absolute absence. In cucumber plants, it has been shown that a low Fe dose of 2–5 µM is necessary to obtain adequate FCR ratios in chlorotic plants. Moreover, any Fe deposit on the root surface should be avoided by using an additional amount of free ligand. Metal buffered solutions with EDTA as described by Cieschi and Lucena [44] are good options to avoid metal depositions on the root surfaces. This EDTA ligand must not contain any Fe, and all the reagents employed in the assay should be of analytical grade, preventing any undesirable metal interference. A growing period from two to four weeks can be suitable to obtain young chlorotic plants to be subjected to FCR assays. Experiments are recommended to start at least two hours after the daylight period to ensure that the normal reduction mechanism is active. Before submerging the excised apical roots or the complete roots in the Fe treatment, the roots must be washed until the Fe is completely absent from the root surface. With this purpose in mind, washing them in a solution with the trapping ligand is required [19]. Then, plants should be transferred to aerated covered pots, preventing the solution from exposure to any light. The test solution must contain a pH-buffered macronutrient solution, avoiding any pH change due to root acidification, and the Fe^2+^ trapping agent (BPDS being the most generalized compound in the literature). Other metallic micronutrients should not be added. Most studies regarding FCR assays use a pH of 6.0 or even lower, because of the optimal pH for the FCR activity. In these cases, an organic MES buffer should be added. However, it must be considered that Fe chlorosis is prevalent in alkaline growing conditions; thus, the FCR assays at a higher pH such as 7.5 (buffered with HEPES) would be more reliable when studying the capacity of the FCR enzyme-reducing Fe from Fe fertilizers in calcareous soil conditions. Then, the Fe fertilizers should be added to the growing media, and aliquots should be sampled several times (e.g., 0, 10, 20, 60, and 120 min), and stored in the dark for further spectrophotometric measurements. Spectrometric determinations must be conducted without much delay to avoid any chemical interaction or degradation. For Fe(BPDS)_3_, the maximum absorbance is obtained at 535 nm. A scheme of the experimental design is shown in Figure 2.

A standard curve for the Fe(BPDS)_3_ must be prepared under the same condition and its slope must be used for the calculations of the Fe(II) chelated by this chelating agent. Alternatively, the molar coefficient of 22.14 mM^−1^ cm^−1^ already described in the literature can be used. This is commonly carried out when chelates such as EDTA/Fe^3+^ do not absorb at 535 nm. However, most of the Fe fertilizers used in agriculture present absorbance at 535 nm, so this contribution should be considered. As an example, EDDHA/Fe^3+^ has a strong absorbance peak at 480 nm with a shoulder that reaches over 535 nm. In this case, the absorbance at 480 nm has to be also recorded in all the samples and the determination of the concentrations of Fe reduced and chelated by BPDS ([Fe(II)(BPDS)_3_]) can be estimated by solving a two equations system (Equations (1) and (2)) [19]:

A535 = aFe(II)BPDS535 × [Fe(II)(BPDS)_3_] + aFe(III)EDDHA535 × [EDDHA/Fe^3+^] (1)

A480 = aFe(II)BPDS480 × [Fe(II)(BPDS)_3_] + aFe(III)EDDHA480 × [EDDHA/Fe^3+^] (2)

where A535 and A480 are the absorbances measured for each sample at 535 and 480 nm, respectively; aFeBPDS535, aFeBPDS480, aFeEDDHA535, and aFeEDDHA480 are the molar absorption coefficients determined from each chelate standard curves at the two wavelengths. With this method, the concentration of the remaining chelate ([EDDHA/Fe^3+^]), not reduced, is also quantified, allowing the mass balance calculation of the [Fe(BPDS)_3_] + [EDDHA/Fe^3+^], which should match the initial concentration of the Fe chelate applied. Other Fe fertilizers, different from synthetic Fe chelates, such as Fe complexes of LS, LN, or HG may also require determining the absorbance at 600 nm [25], a characteristic wavelength for these compounds.

The use of a pair of plants instead of one in each container (representing one replicate) improves the confidence of the method, reducing heterogeneity frequently found in biological assays with young plants, and including at least seven replicas per treatment. Additionally, at least two replicates without the Fe treatment must be included as controls.

When the treatments under study are expected to have any impact on the root excretion of reductants, the contribution to the Fe reduction by these compounds can be quantified by including additional pots without the addition of BPDS during the period of incubation but adding it just before the analysis of the absorbance [27].

However, it must be noted that this methodology has not been shown to be suitable enough for all Fe products, especially those Fe complexes with large molecular weights and variable structures. Cieschi and Lucena [44] observed a low Fe reduction rate by FCR in two hours of assay when an LN/Fe^3+^ was used as the FCR substrate in stressed cucumber plants. These complexes have been demonstrated to release Fe to the plant in the long term. Similarly, Martín-Fernández et al. [25] did not observe FCR activity for other Fe complexes in chlorotic soybean plants. Therefore, when the Fe sources object of the study are products characterized by a slow release, the study of the Fe acquisition throughout the FCR activity would not be suitable.

Because it has been shown that the plant does not assimilate all the reduced Fe by the FCR (see Section 4.1), it would be necessary to complement the FCR assay with an Fe uptake experiment. The use of isotopically labeled Fe sources to determine the Fe resupply with plants grown under identical conditions would be recommended. The use of the stable isotope ^57^Fe as a tracer is widespread for monitoring Fe in plant organs, both in hydroponics and in soil [21,29,81,82], and it is a valuable tool for assessing the effectiveness of different Fe fertilizers [83].

**Figure 3:**
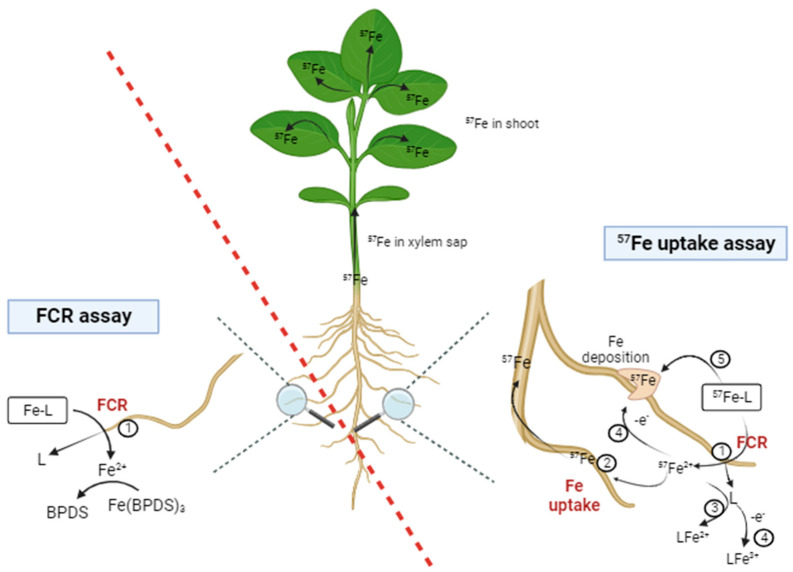
Conceptual scheme describing the chemical processes occurring during the FCR assay (left) and the ^57^Fe uptake assay (right): ➀ Fe(III) reduction to Fe(II) by FCR enzyme, ➁ Fe uptake and translocation into the plant, ➂ Fe^2+^ chelation by free ligand released after the FCR Fe reduction, and ➃ reoxidation of L-Fe^2+^ to L-Fe^3+^, and ➄ Fe^3+^ deposition in roots. Created with BioRender.com.

The second proposed methodology includes a parallel and similar experimental setup to the one described for the FCR tests, with two main differences. A conceptual figure describing the different chemical processes occurring during the FCR and Fe uptake assays proposed is shown in Figure 3. It must be noticed that although other compounds are involved in Fe uptake in Strategy I, such as organic acids, flavins, and phenolic [33], Figure 3 only shows the main aspects related to the tests for Fe reduction by FCR and Fe uptake in the solution.

After a growing period to obtain moderate chlorotic plants, they are transferred to aerated pots containing a nutrient macronutrient solution buffered at the desired pH (MES for pH 6 and HEPES for pH 7.5) avoiding any pH change due to root acidification, similar to that described before for the FCR assay. But, for the Fe uptake assay, the Fe^2+^ trapping agent is not used. At time 0, the Fe treatments labeled with ^57^Fe should be similarly added. In this case, only one sampling time is considered—after 2 h, a period that must be enough to observe the Fe translocation into the different plant organs in common Fe fertilizers. The whole plant is kept for further analysis; for that, it must be washed, and roots, stems, and leaves must be separated and weighed. Since in the FCR assay, the reduced Fe^2+^ is trapped in the solution for its determination, Fe does not enter the plant, and thus, the plant material is not analyzed any further. Only the root weight is recorded to express the FCR activity in units referred to as fresh root weight. After that, the plant material is wasted. For the Fe uptake assay, the dried plant material (usually after two days at 65 °C) is mineralized and digested, and the Fe isotope (54, 56, 57, and 58) concentrations are determined by using an ICP-QMS. A mathematical deconvolution should be made to differentiate the two Fe sources, one coming from the fertilizer (named Fe_fer_) and the other coming from natural origin (named Fe_nat_, i.e., seeds, growing media) [79]. The results obtained provide a better understanding of the processes that the Fe has undergone from the fertilizer and its localization in the plant. Stem and leaf contents are related to the complete reduction and uptake process. If a large amount of Fe_fert_ is found in the root, it must partially be attributed to the formation of Fe deposits on the roots.

Following this methodology, Nadal [65] studied the relationship between Fe reduced by FCR and Fe absorbed as ^57^Fe by plants, using cucumber (*Cucumis sativus* L. cv. ‘Ashley’) as an efficient Strategy I plant model and soybean (*Glycine max* L. cv. Speeda) as a non-efficient Strategy I plant model at the optimal pH for the FCR, pH 6.0. In parallel, the Fe concentration in the xylem sap was analyzed in their experiment (Table 4). The results obtained compared two known Fe chelates, finding that both plant species reduced around three times more Fe when the Fe source for the FCR was EDTA/Fe^3+^ than when *o,o*EDDHA/Fe^3+^ was used. Moreover, the assimilated Fe determined by the ^57^Fe uptake assay was much lower than the reduced Fe. All the discussed results confirmed the validity of the method proposed by combining both experiments, FCR assay, and Fe uptake assay, conducted in parallel, for a better understanding of the Fe reduction by FCR and the subsequent uptake and translocation into the plant. It is possible to identify other possible reactions occurring in the nutrient solution such as Fe reoxidation and deposition in roots, and the chelation of the FCR reduced Fe^2+^ via a free ligand. Thus, the combination of both assays will provide fundamental information about the mechanism involved in the interaction between the fertilizer and the plant, independently of the soil interaction with the fertilizer.

## 6. Conclusions

The reduction of Fe(III) to Fe(II) is a fundamental step in the acquisition of Fe in Strategy I plants. This review points out the factors that affect the FCR activity, including plant species, pH of the medium, Fe nutritional plant status, Fe substrate, and *in vivo* or *in vitro* conditions. The Fe substrate has been shown to be a determining factor in the FCR efficiency, which is affected by chemical properties such as the Fe(III)-L and Fe(II)-L stability, ligand structure, polarity, and the presence and type of functional groups able to bind Fe, among others. Despite the large number of variables concerning FCR activity, this assay provides relevant information on plant responses to Fe fertilizers. However, the study of the Fe acquisition of new Fe deficiency treatments needs to be completed with other information about the interaction between the fertilizers and the plant, including an Fe uptake assay. The proposed methodology combines the two assays, the FCR activity, and the Fe uptake. The incorporation of the isotopic labeling, such as the stable isotope ^57^Fe, into the Fe uptake assay will additionally contribute to a better distinction between the Fe uptake and translocated from the Fe fertilizer. Experiments conducted with this combined methodology provide information on both the Fe uptake and reduction process, allowing a better understanding of the efficacy of Fe fertilizer to give Fe to the Strategy I plants.

## Figures and Tables

**Table 3:** Summary of selected thermodynamic stability constants (logK^0^_ML_) of the synthetic Fe chelates used as substrate in the FCR activity assay and the publication in which the reduction assay was studied.

[ML]/[M][L]	logK^0^	FCR Assay
*o,o*EDDHA	37.7	[21,22,23,25,26,27,44,65,66,67]
meso *o,o*EDDHA	36.7	[19,68]
rac *o,o*EDDHA	38.4	[19,68]
*o,p*EDDHA	31.3	[22,66]
EDDHSA	36.6	[19]
HBED	42.2	[19,21]
HJB	36.4	[21]
DCHA	29.9	[22]
EDTA	27.6	[19,25,26,27,28,65,66]
[*S,S*]-EDDS	23.7	[25]
IDHA	16.4	[25]

Note: logK^0^ values were directly obtained from [69] except for EDTA which was calculated from log K_c_ obtained by [70].

**Table 4:** FCR assay and ^57^Fe uptake assay for Fe resupply (µmol h^−1^ g^−1^ root fresh weight) in cucumber and soybean plants. Data values are means ± (SE) (adapted from Nadal [65]).

	EDTA/Fe^3+^	*o,o*EDDHA/Fe^3+^
FCR activity		
Cucumber	4.08 ± 0.46 ns (A)	1.44 ± 0.13 a (B)
Soybean	3.82 ± 0.36 (A)	0.92 ± 0.11 b (B)
^57^Fe xylem sap		
Cucumber	0.009 ± 0.001 ns (B)	0.013 ± 0.001 ns (A)
Soybean	0.008 ± 0.001 (B)	0.011 ± 0.001 (A)
^57^Fe shoot		
Cucumber	0.009 ± 0.001 ns (NS)	0.012 ± 0.004 ns
Soybean	0.006 ± 0.001 (B)	0.017 ± 0.003 (A)

Note: Lowercase letters in the same column denote significant differences between plant species for each Fe treatment (Duncan Test, *p* < 0.05). Uppercase letters in rows denote significant differences between treatments for each plant species (*p* < 0.05). NS or ns denotes no significant difference.

## Data Availability

All the data used for this manuscript have been already published in the literature and properly cited in the text.
